# Bilateral Posterior Four-Part Fracture-Dislocation of the Proximal Humerus After First-Time Seizure

**DOI:** 10.7759/cureus.17688

**Published:** 2021-09-03

**Authors:** John D Murphy, Phillip R Braunlich, Mohit Bansal, Lauren Edge, John N Harker

**Affiliations:** 1 Orthopaedic Surgery, Largo Medical Center, Largo, USA

**Keywords:** proximal humerus fracture, proximal humerus fracture-dislocation, reverse total shoulder arthroplasty, rtsa, seizure, posterior dislocation of the shoulder, bilateral proximal humerus fracture, bilateral shoulder fracture-dislocation, four part fracture-dislocation, bilateral shoulder arthroplasty

## Abstract

This report presents a previously undescribed case and treatment of bilateral four-part proximal humerus (PH) fracture-dislocations presented in a 61-year-old Caucasian male patient following a first-time seizure episode. The patient was treated with bilateral reverse total shoulder arthroplasty due to pre-existing glenohumeral arthritis and rotator cuff atrophy. The surgery was successful, and the patient’s postoperative recovery was uneventful. Fractures of the proximal humerus are a relatively common adult osteoporotic fracture; however, posterior fracture-dislocations of the PH, frequently related to motor vehicle accidents, seizures, or electrical shock, are remarkably scarce. A treatment algorithm for these injuries is lacking.

## Introduction

Proximal humerus fractures, often classified as osteoporotic fractures due to an average age of occurrence of 64.8 years, account for approximately 5.7% of adult fractures and occur over twice as often in females [1]. Posterior shoulder dislocations are rare, comprising approximately 2-5% of all shoulder dislocations [2]. Bilateral posterior shoulder dislocations include less than 5% of all posterior shoulder dislocations [3]. Compared to isolated proximal humerus (PH) fractures, fracture-dislocations of the PH occur in only 1% of all shoulder dislocations, making PH fracture-dislocations exceedingly rare [4]. Posterior dislocations are often associated with motor vehicle accidents, seizures, or electrical shock [5].

As with most fractures, PH fractures can be treated non-operatively, but if the greater tuberosity part is displaced greater than 5mm, open reduction and internal fixation (ORIF) is indicated. Other operative approaches include closed reduction and percutaneous pinning (CRPP), intramedullary nailing (IMN), hemiarthroplasty (HA), anatomic total shoulder arthroplasty (aTSA), and reverse total shoulder arthroplasty (rTSA) [4].

Complications of PH fractures include neurovascular injury, avascular necrosis, hardware failure, and nonunion [4]. The axillary nerve exits the quadrangular space posteriorly, traversing horizontally from medial to lateral around the posterior humerus, and is located approximately 5 to 7 cm distal to the posterior edge of the acromion [4]. It is at risk with the placement of lateral K-wires during CRPP. The factors that indicate a higher risk of humeral head ischemia are described by the Hertel criteria, which include the following: part displacement greater than 10mm, part angulation greater than 45 degrees, disruption of the posterior-medial hinge, a diaphyseal extension less than 8mm, and increasing fracture complexity [4].

## Case presentation

**Figure 1:**
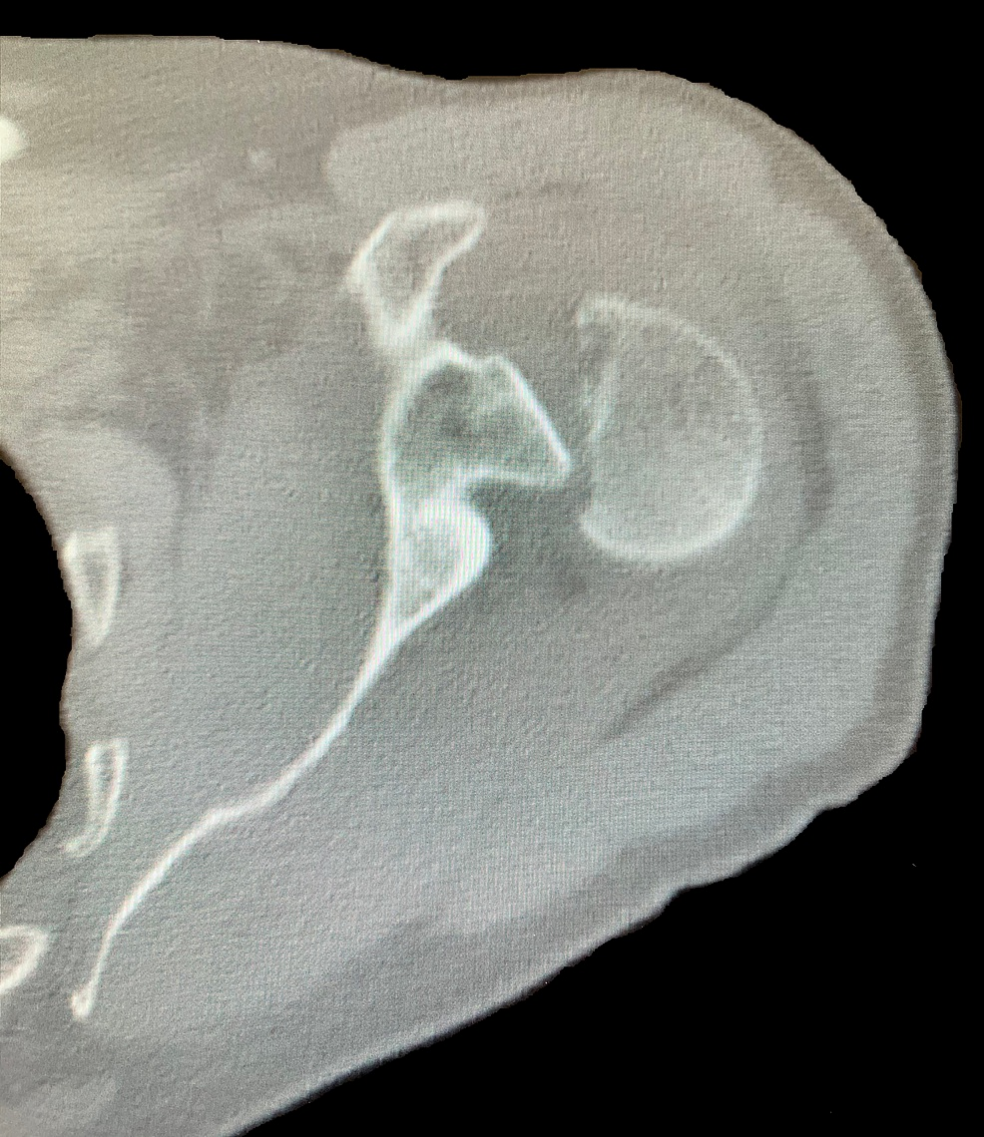
Axial view of preoperative computed tomography (CT) scan of the left shoulder demonstrating a four-part proximal humerus (PH) posterior fracture-dislocation.

**Figure 2:**
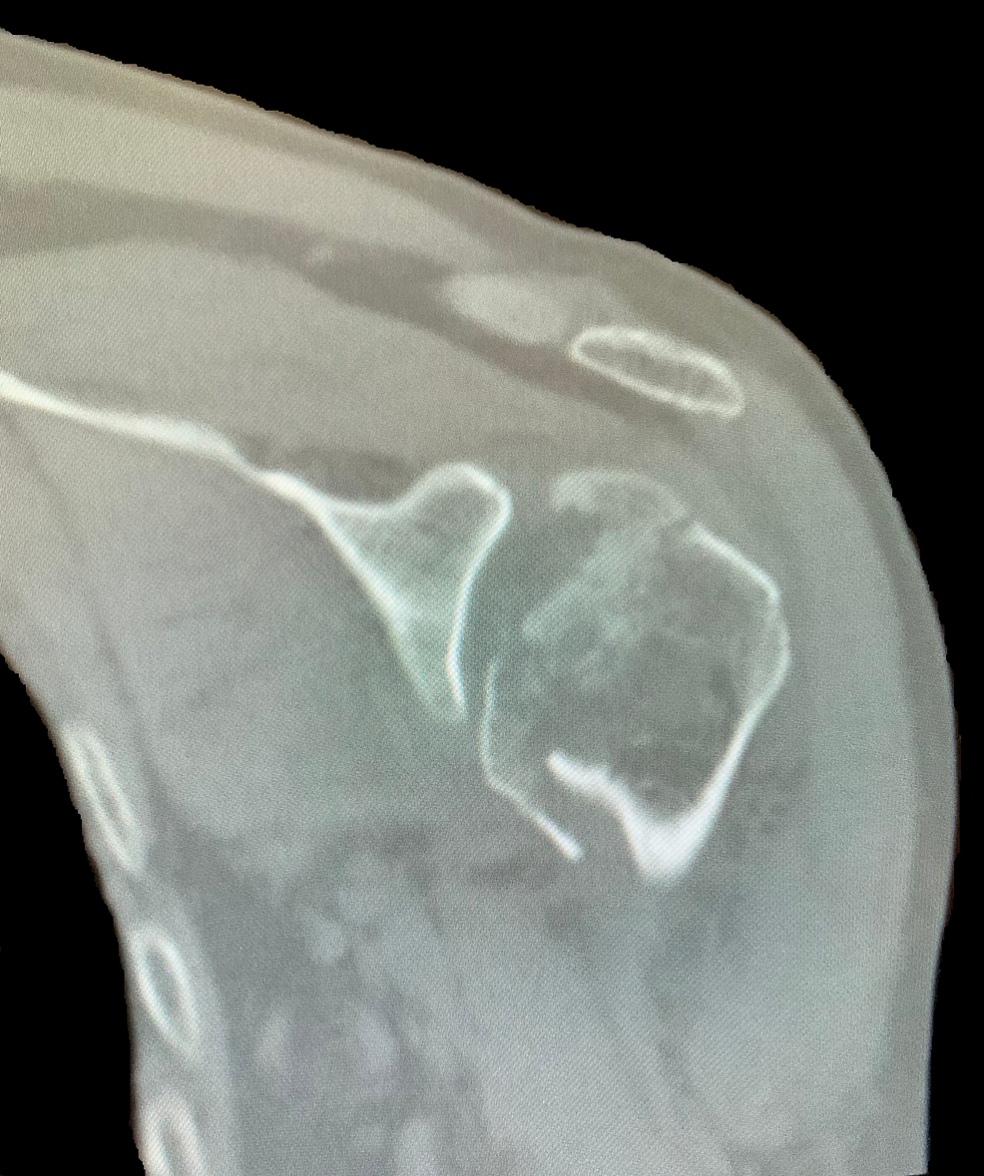
Coronal view of preoperative CT scan of the left shoulder demonstrating a four-part proximal humerus (PH) posterior fracture-dislocation.

**Figure 3:**
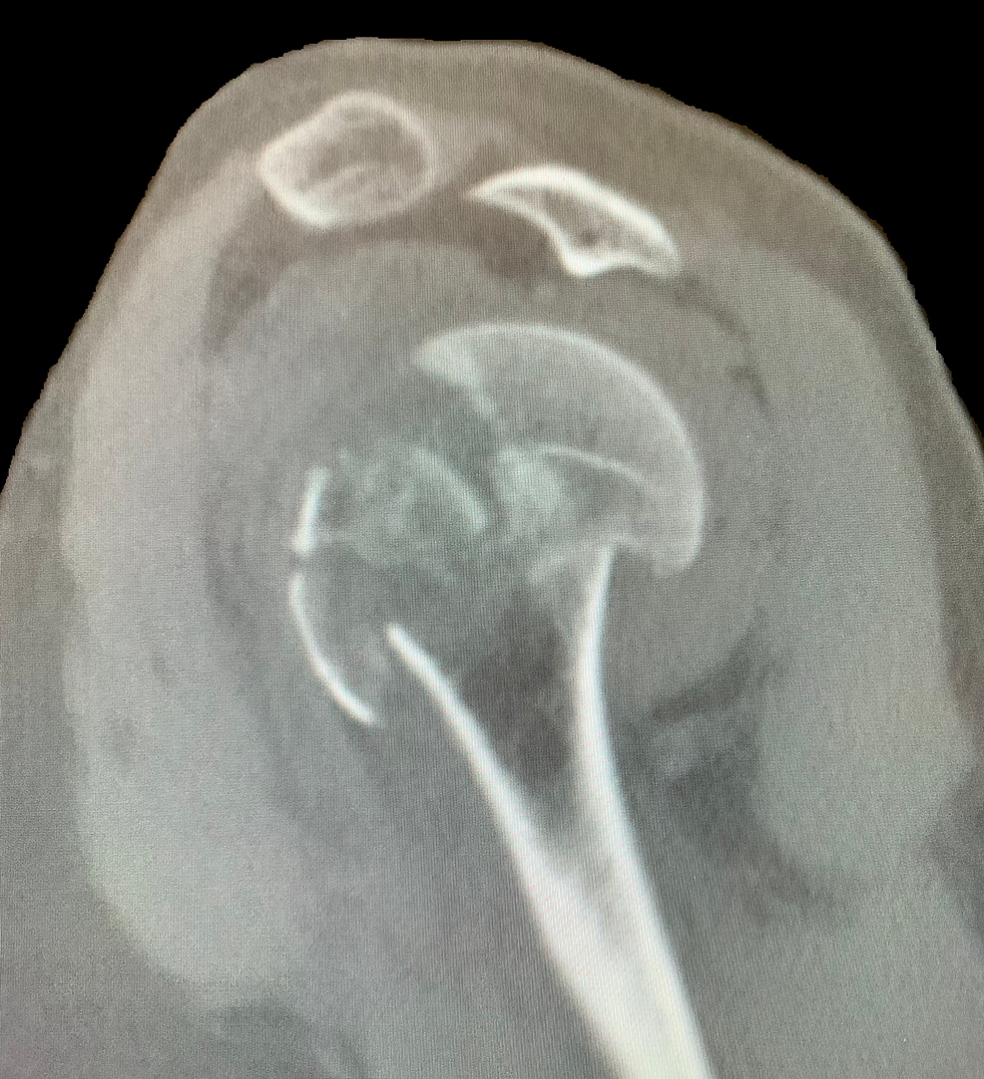
Sagittal view of preoperative CT scan of the left shoulder demonstrating a four-part proximal humerus (PH) posterior fracture-dislocation.

**Figure 4:**
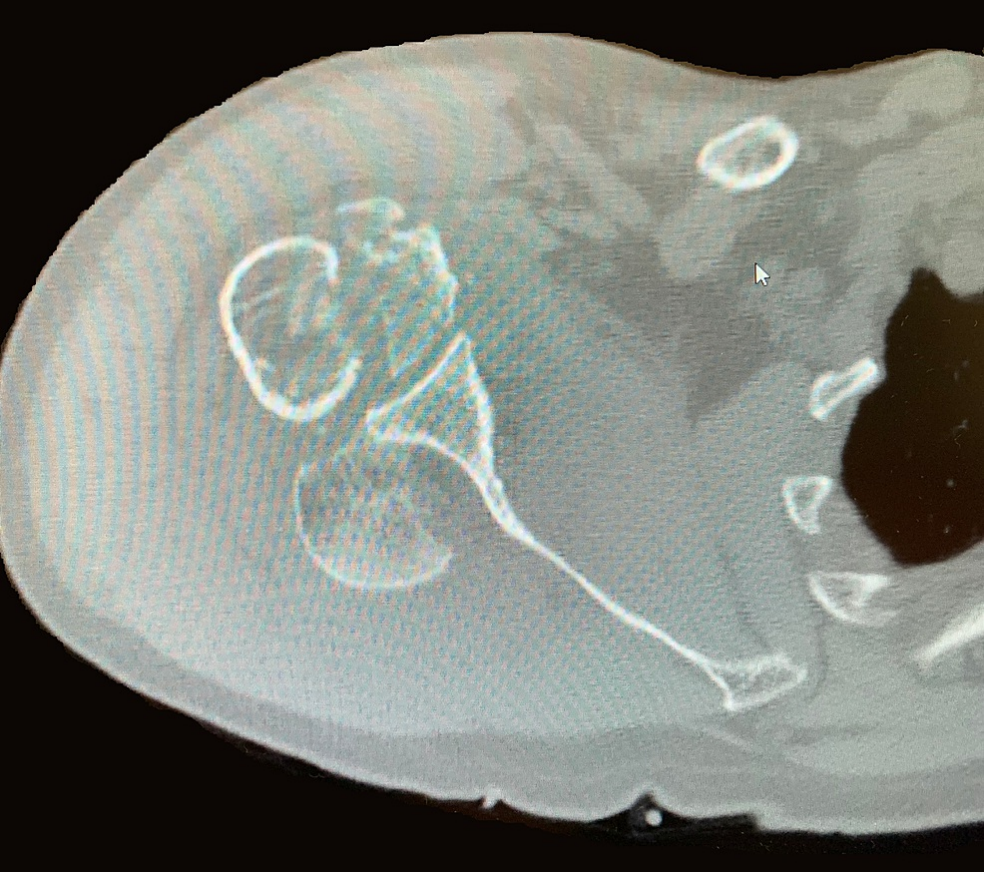
Axial view of preoperative CT scan of the right shoulder demonstrating a four-part proximal humerus (PH) posterior fracture-dislocation.

**Figure 5:**
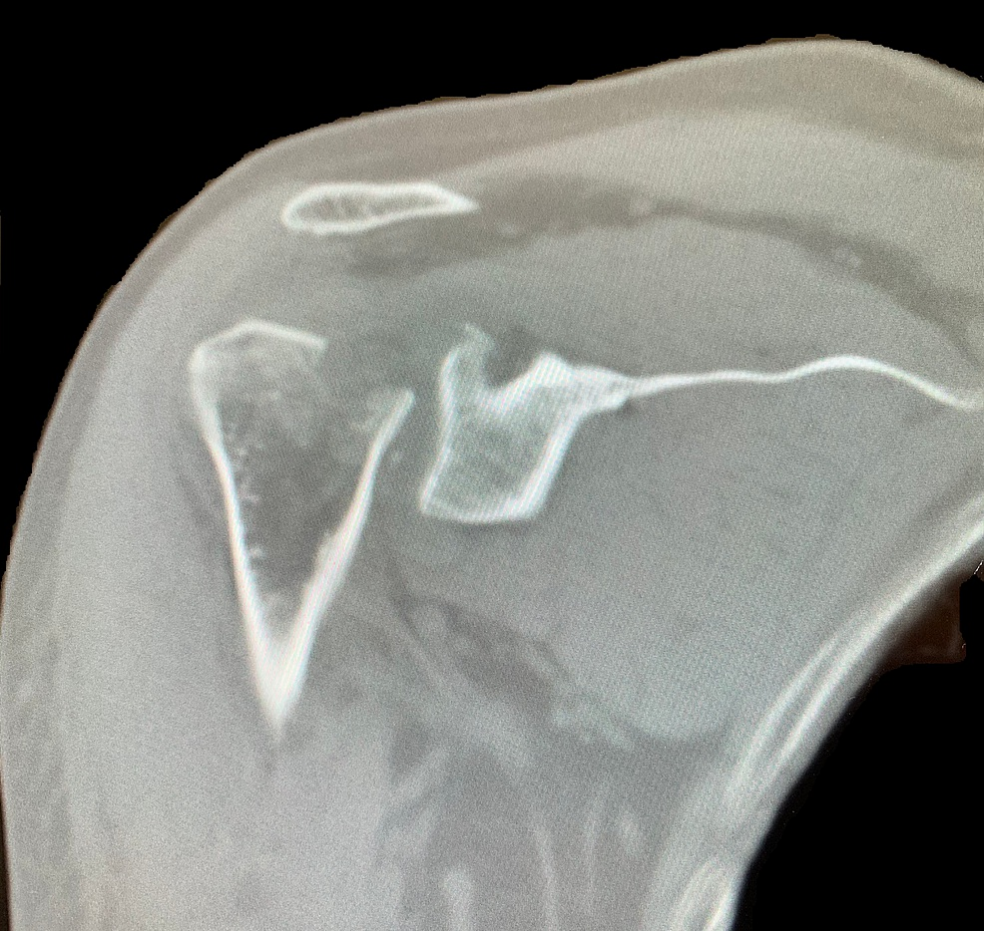
Coronal view of preoperative CT scan of the right shoulder demonstrating a four-part proximal humerus (PH) posterior fracture-dislocation.

**Figure 6:**
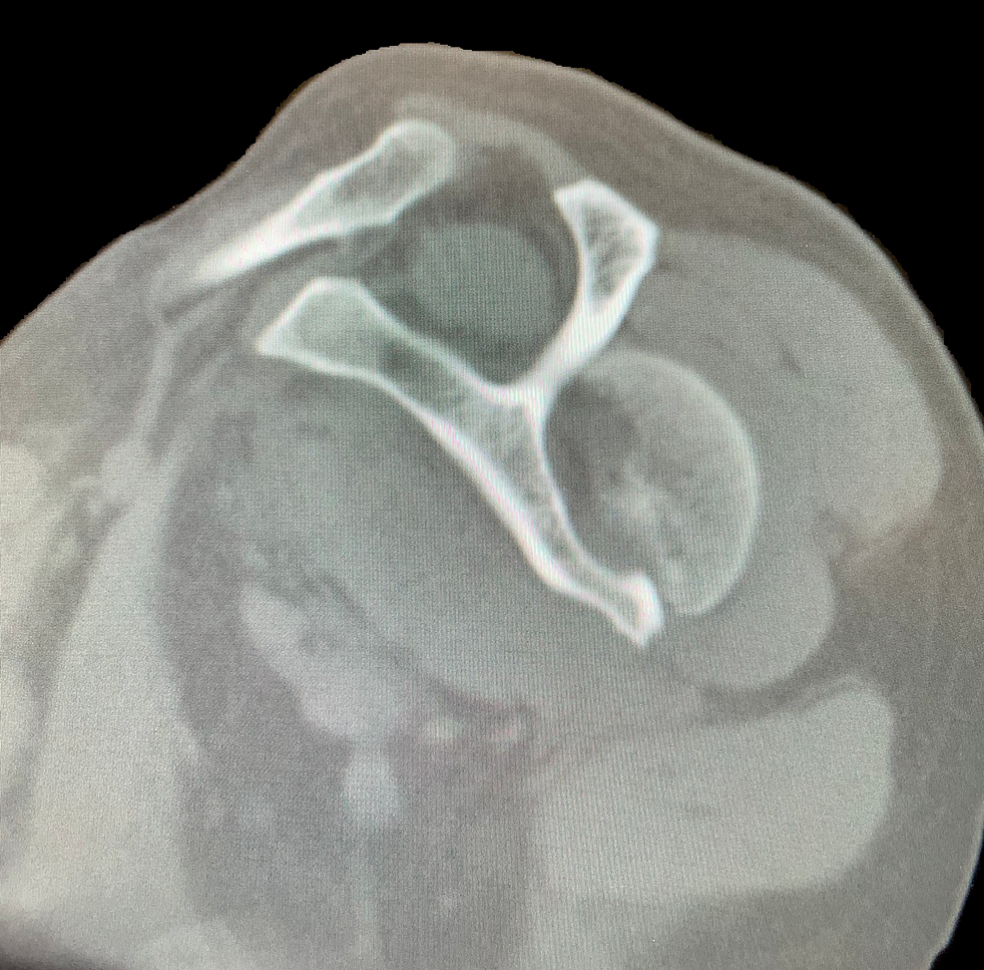
Sagittal view of preoperative CT scan of the right shoulder demonstrating a four-part proximal humerus (PH) posterior fracture-dislocation.

A 61-year-old Caucasian male presented to a Costa Rican hospital after suffering a grand mal seizure while on vacation. After his seizure evaluation, the patient flew back to the United States. Two days after the seizure, he presented to a local Veterans Affairs hospital. The patient’s medical history was significant for chronic obstructive pulmonary disease (COPD), gastroesophageal reflux disease (GERD) on a daily proton pump inhibitor (PPI), hypertension (HTN), previous alcohol dependence, tobacco use, and no illicit drug use. He did not have a history of seizures. He was found to have hyponatremia of 128 mg/dl. The patient was also complaining of decreased shoulder range of motion and pain bilaterally. Radiographs demonstrated bilateral, four-part fracture-dislocations of the proximal humerus (PH) with severe comminution. The patient was placed in bilateral slings and instructed to follow up with an orthopedic surgeon. After orthopedic surgery evaluation, it was determined the patient would require bilateral reverse total shoulder arthroplasty. Computed tomography (CT) scans were obtained of the bilateral shoulders, demonstrating bilateral four-part fracture-dislocations of the proximal humerus, as seen in Figures 1-6.

**Figure 7:**
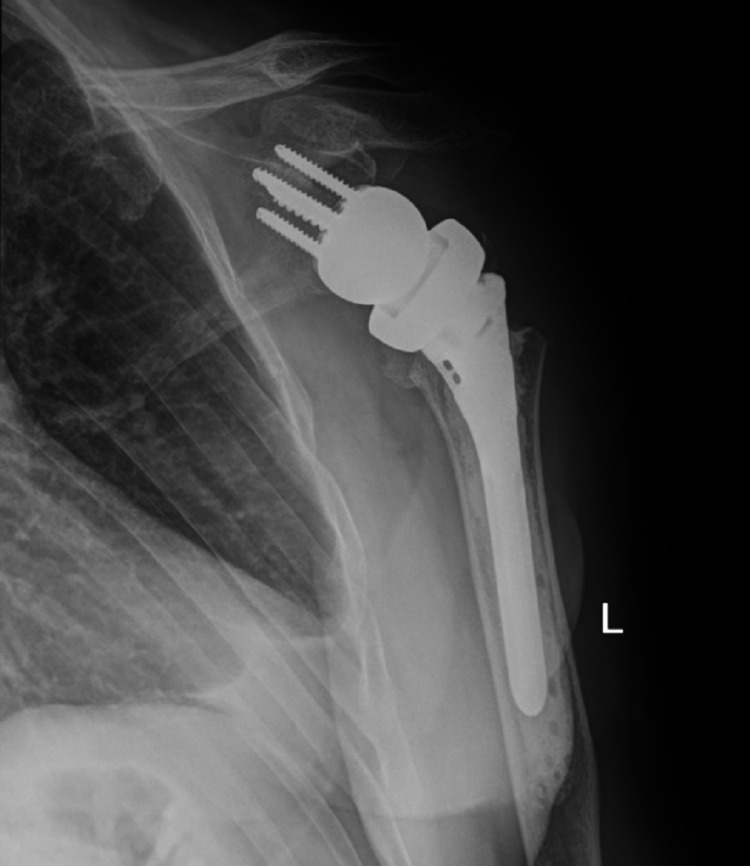
AP postoperative radiograph of the left shoulder at 12 weeks demonstrates a reverse total shoulder arthroplasty (rTSA) in satisfactory alignment without signs of loosening or failure.

**Figure 8:**
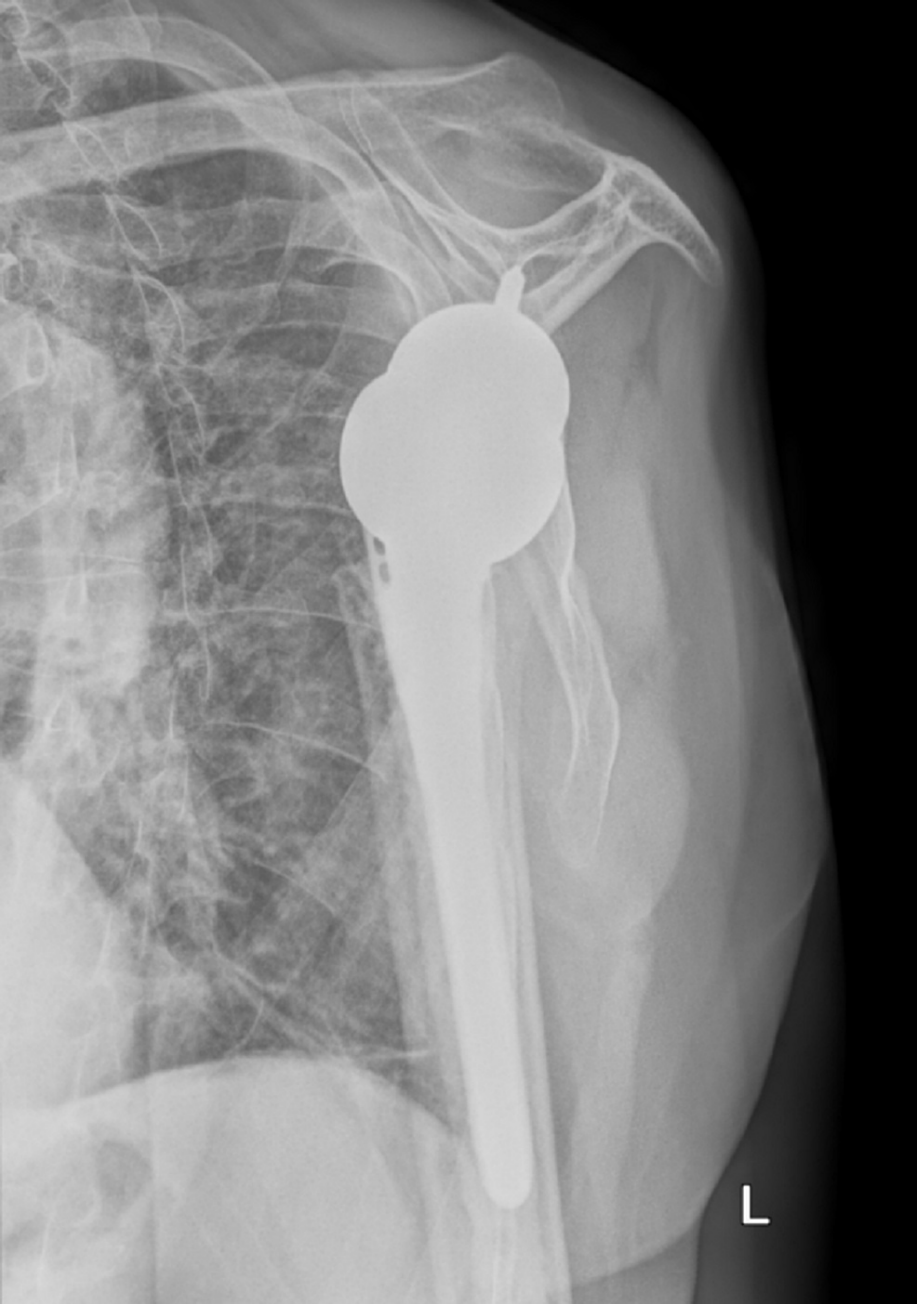
Scapular Y postoperative radiograph of the left shoulder at 12 weeks demonstrates a reverse total shoulder arthroplasty (rTSA) in satisfactory alignment without signs of loosening or failure.

**Figure 9:**
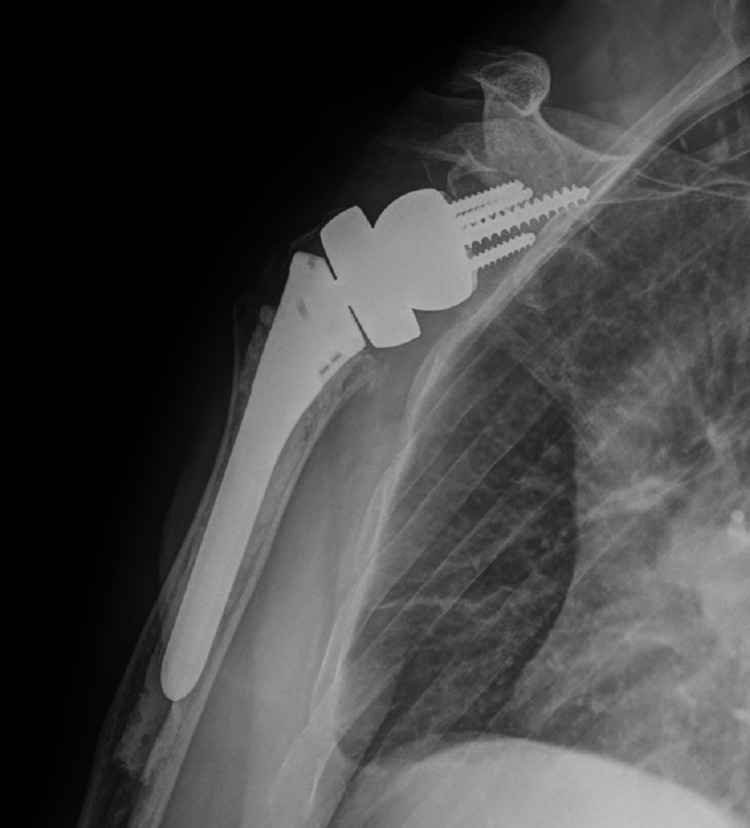
AP postoperative radiograph of the right shoulder at 12 weeks demonstrates a reverse total shoulder arthroplasty (rTSA) in satisfactory alignment without signs of loosening or failure.

**Figure 10:**
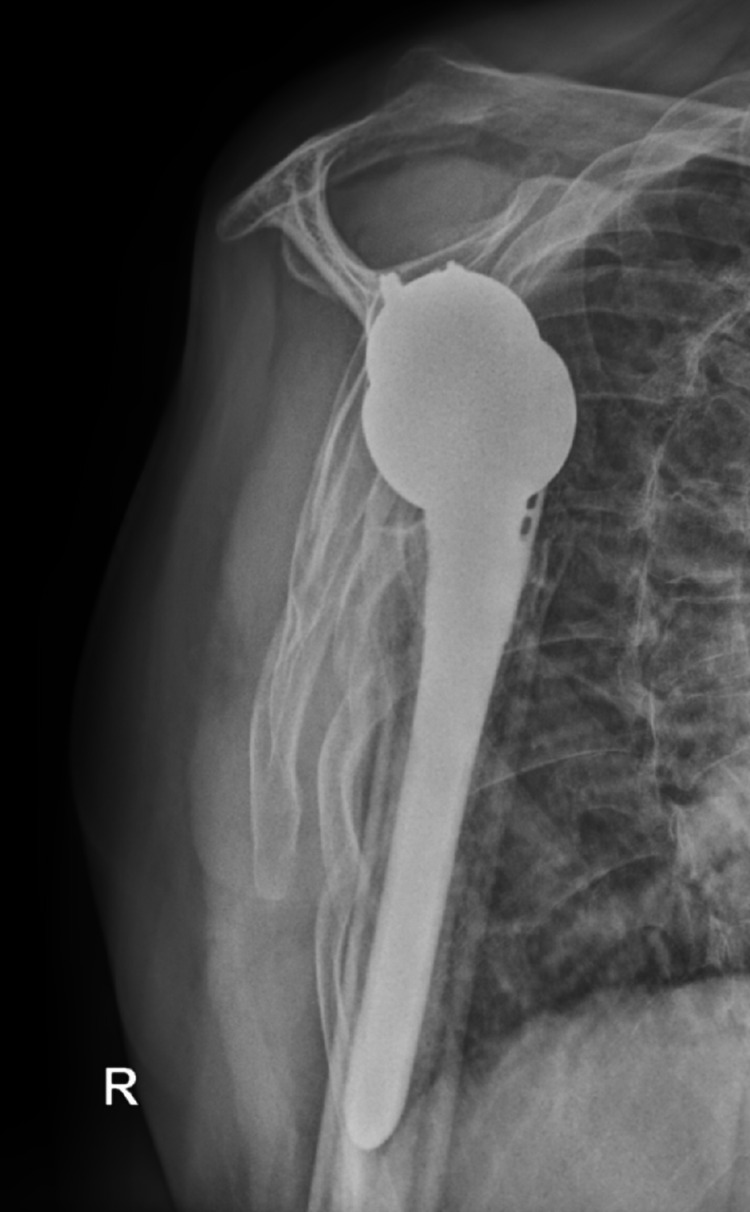
Scapular Y postoperative radiograph of the right shoulder at 12 weeks demonstrates a reverse total shoulder arthroplasty (rTSA) in satisfactory alignment without signs of loosening or failure.

The decision to proceed with bilateral reverse total shoulder arthroplasty was made because the patient’s rotator cuffs were retracted and atrophied. He had also been experiencing a significant amount of glenohumeral arthritis before the seizure. He underwent staged reverse total shoulder arthroplasties, the first being the right side, his dominant hand, which was performed six days post-injury. The subsequent second stage was performed on the left side 10 days post-injury. He tolerated both procedures well and had an uneventful postoperative recovery. The patient completed physical therapy programs for both shoulders, and he was compliant with all follow-up appointments. At a one-year follow-up, the patient’s range of motion was equal in both shoulders: forward flexion to 120 degrees, abduction to 120 degrees, external rotation to 50 degrees, and internal rotation to L4. He was very pleased with his operative outcome. Postoperative radiographs of the bilateral shoulders obtained at 12 weeks postoperatively are seen in Figures 7-10. These images demonstrate bilateral reverse total shoulder arthroplasties in satisfactory alignment without evidence of loosening or hardware failure.

It is unclear whether daily PPI use, chronic alcohol use, and/or tobacco use put the patient at risk for fracture, but these may have been contributing factors. The etiology of his seizure was also unclear but may have been associated with the reported seizure threshold lowering effects of PPIs [6].

## Discussion

The purpose of this paper is to discuss the rare occurrence of a patient presenting with bilateral, four-part fracture-dislocations of the proximal humerus (PH), which was treated with bilateral reverse total shoulder arthroplasty. For this case, the patient suffered four-part fracture-dislocations which are associated with a higher rate of avascular necrosis of the humeral head, he also had preexisting glenohumeral arthritis in both shoulders, and he had a delayed presentation [7]. For these reasons, it was determined the patient would benefit from reverse total shoulder arthroplasty. Upon literature review, nine unique cases of bilateral PH fracture-dislocations were described [8-16]. As indicated, the etiology of the bilateral PH fracture-dislocations included electrocution, first-time seizure, second-time seizure, and multiple seizures. The patient ages ranged from 29 years old to 69 years old, with a mean age of 50.5 years old. Patterns for the fractures included bilateral one-part fracture-dislocation, bilateral two-part fracture-dislocation, bilateral three-part fracture-dislocation, unilateral two-part fracture-dislocation with ipsilateral four-part fracture-dislocation, and one undefined fracture pattern. Treatment methods included bilateral non-operative treatment with closed reduction, closed reduction with percutaneous pinning, unilateral non-operative treatment with contralateral shoulder hemiarthroplasty (HA), unilateral open reduction internal fixation (ORIF) with contralateral non-operative treatment, bilateral ORIF, unilateral ORIF with contralateral shoulder HA, bilateral cemented shoulder HA, and initial treatment with closed reduction that was lost to follow-up for definitive treatment. There were no occurrences found of bilateral, four-part fracture-dislocations of the PH, or of bilateral reverse total shoulder arthroplasty having been used to treat bilateral, four-part fracture-dislocations of the PH.

Based on the patient’s successful postoperative course, we would have not proceeded with non-operative treatment, ORIF, hemiarthroplasty, or anatomic shoulder arthroplasty. Staging of the procedure allowed for adequate post-operative recovery and early passive range of motion. This is a novel case of bilateral four-part proximal humerus fracture-dislocations treated with staged reverse total shoulder arthroplasties, of which no similar presentation was found during literature review. It is imperative to recall the association between seizure and posterior glenohumeral dislocations. While numerous treatment options are at the surgeon’s disposal, reverse shoulder arthroplasty provides a predictable outcome, especially in patients with delayed presentation and pre-existing cuff tear arthropathy.

## Conclusions

In patients with rare-occurring bilateral four-part proximal humerus fracture-dislocations in the setting of bilateral chronic arthritis and rotator cuff tear arthropathy, bilateral reverse shoulder arthroplasty was shown to be a feasible treatment option to restore functional range of motion and long-term pain relief. There is potential to extrapolate the success of this case in the future when weighing treatment options for differing mechanisms of injury and fracture patterns of the proximal humerus requiring surgical fixation.
